# Comprehensive analysis of plasma exosomal and non-exosomal microRNA profiles identifies potential early indicators of carcass traits in Japanese Black cattle

**DOI:** 10.1186/s12864-026-12820-w

**Published:** 2026-04-06

**Authors:** Kevin Otieno Amolo, Shota Inagaki, Yuya Adachi, Zeng Yang, Shinichiro Ogawa, Norihide Yokoi

**Affiliations:** Laboratory of Animal Breeding and Genetics, Division of Applied Biosciences, Graduate School of Agriculture, Kyoto University, Kitashirakawa Oiwake-Cho, Sakyo-Ku, Kyoto, 606-8502 Japan

**Keywords:** Blood biomarkers, Carcass traits, Exosomal microRNAs, Japanese Black cattle, Non-exosomal free circulating microRNAs

## Abstract

**Background:**

Plasma microRNAs (miRNAs), which exist in exosomal (EX) and non-exosomal free circulating (FC) fractions, can be utilized as blood biomarkers. To identify early indicators of carcass traits, we performed comprehensive analysis of plasma miRNAs separately on EX and FC fractions in Japanese Black cattle at 20, 25, and 30 months of age.

**Results:**

We found a total of 1,071 miRNAs including 39 novel miRNA candidates, in which 345 and 358 miRNAs exhibited in measurable amounts in EX and FC fractions, respectively. Profiles of these miRNAs were similar at 20 and 25 months, but collectively different from those at 30 months. We identified 86 and 80 differentially existed miRNAs (DE-miRNAs) between 25 and 30 months in EX and FC fractions, respectively. Among the DE-miRNAs, we revealed significant correlations of *bta-miR-150* (EX and FC), *-30b-5p* (EX), *-423-3p* (EX), and *-222* (FC) with several carcass traits including marbling score. Interestingly, target genes of these DE-miRNAs were functionally enriched in KEGG pathways such as PI3-Akt and MAPK signaling pathways, suggesting their functional relevance to carcass quality and quantity traits.

**Conclusions:**

We clarified changes in plasma EX and FC miRNA profiles during the fattening period and identified several miRNAs having correlations with carcass traits. These results could support the utility of plasma miRNAs as potential early indicators of carcass traits in Japanese Black cattle.

**Supplementary Information:**

The online version contains supplementary material available at 10.1186/s12864-026-12820-w.

## Background

Japanese Black cattle are fattened for relatively longer periods (~ 30 months of age) to maximize beef marbling and other carcass traits. However, long fattening period often results in high feed consumption and feed wastage due the lower feed efficiency especially during late fattening phase, thus contributing to high production cost of Japanese Black cattle [1]. It has been reported that several steers exhibit excess level of intramuscular fat content at younger ages [2], raising the possibilities of shortening the fattening period in Japanese Black cattle while maintaining the beef quality traits [1, 3]. Previous study has suggested that shortening the fattening period can reduce feed consumption without affecting the beef quality in Hanwoo cattle [4]. Because major carcass traits can only be observed after slaughter [5], decision of early slaughter would require early indicators of beef quality and quantity status in live Japanese Black steers.

MicroRNAs (miRNAs) are short, non-coding RNAs of approximately 19–23 nucleotides which regulate expression of multiple target genes at the post-transcriptional level [6, 7]. A variety of miRNAs have been detected in plasma as a result of passive or active leakages from cells expressing them. These miRNAs can be packed within exosomes (exosomal miRNAs), as well as be bound to plasma protein complexes (non-exosomal miRNAs). Plasma microRNAs are stable and can reliably be delivered from their donor cells to modulate the physiological state of the related recipient cells [8, 9] and their profiles potentially reflect the physiological state of various tissues of their origin [10–12]. To date, several studies have demonstrated the potential of plasma miRNAs as biomarkers for various physiological traits in cattle. For instance, plasma miRNAs are associated with early life performance and aging in dairy cattle [13, 14]; miRNAs in plasma small extracellular vesicles as potential early indicators of dairy cow subfertility [15]; the potential regulatory role of blood miRNAs in various lameness phenotypes in feedlot cattle [16]; blood microRNAs as potential candidate biomarkers in a subacute ruminal acidosis in Holstein–Friesian cows [17]; the potential of plasma miRNAs as biomarkers for early indicators of pregnancy in Japanese Black cattle [18, 19]. Although there is one study on plasma exosomal miRNA profiling using microarray and qPCR analyses during the fattening in different feeding conditions (indoor grain-feeding vs. grazing on pasture) of Japanese Black cattle [20], no studies have been reported using small RNA sequencing (RNA-seq) to analyze abundance of both plasma exosomal and non-exosomal miRNAs to associate them with carcass traits in Japanese Black cattle.

In the present study, to identify plasma miRNAs that could be potentially used as early indicators of carcass traits, we performed small RNA-seq to analyze plasma exosomal (EX) and non-exosomal free circulating (FC) miRNA profiles, separately during the fattening period of Japanese Black cattle. We identified differentially existed miRNAs (DE-miRNAs) whose abundance varied with age and also found four miRNAs showing significant correlations with carcass traits. These results may support the utility of plasma miRNAs as potential early indicators of carcass traits in Japanese Black cattle.

## Methods

### Animals and sample collection

This study used 28 Japanese Black fattened steers raised under the same feeding system at Kyoto University livestock farm, Kyotambacho, Kyoto, Japan. As with routine blood sampling for health check of the steers, whole blood samples were collected in the morning from jugular veins using Venoject II vacuum blood collection tubes (EDTA-2K) (Terumo) by the well-trained technical staff at the farm without any anesthesia. The blood samples were centrifuged to collect the plasma samples, which were then stored at –80 °C until RNA extraction. Around 30 months of age, all the 28 steers were commercially slaughtered by staff at the Kyoto City Central Wholesale Meat Market according to the standard procedures in Japan. For small RNA-seq, a total of nine plasma samples were collected from 3 out of the 28 steers at approximately 20, 25, and 30 months of age. The age at plasma sampling and carcass performance records at slaughter for the 3 steers are shown in Table S1A and S1B, respectively. For qPCR quantification and correlation analysis of the abundance of plasma miRNAs with carcass traits, we collected plasma samples from all the 28 steers at slaughter age (30.7 ± 1.0 months). For whole genome sequencing, the liver sample was collected from one of the steers just after the slaughter at the market and stored at –80 °C until DNA extraction.

### RNA extraction, small RNA-seq and quality analysis

Both EX and FC RNAs were extracted from 1 mL of plasma samples using Plasma/Serum Exosome and Free-Circulating RNA Isolation Mini Kit (Norgen Biotek) according to the manufacturer’s instructions. This RNA purification is based on spin column chromatography that employs Norgen’s proprietary resin. The RNA isolated from the purified exosomes is free from any protein-bound circulating RNA and is of the highest integrity. Moreover, the free-circulating, protein-bound, RNA is free from any exosomal RNA. Small RNA-seq was performed on the samples at 51 bp single-ended (> 20 M reads/sample) using Illumina NovaSeq 6000 at Azenta Life sciences. The raw data were preprocessed by removing adapter sequences and trimming off low-quality reads using Cutadapt (version 5.0) [21] and Trimmomatic (version 0.39) [22], respectively, retaining high-quality reads of 15–51 bp, which were evaluated using FastQC (version 0.12.1) [https://www.bioinformatics.babraham.ac.uk/projects/fastqc/].

### Generation of a genome sequence of Japanese Black cattle

A whole genome sequence of Japanese Black cattle was constructed through the resequencing approach. Genomic DNA was extracted from the liver of one the steers using Wizard Genomic DNA Purification Kit (Promega) according to the manufacturer’s instructions and the whole genome sequencing was performed at 151 bp paired ends (304 M reads, > 90 Gb) using the Illumina NovaSeq 6000 at Azenta Life sciences. Adapter sequences were removed from the sequencing data, followed by trimming off the low-quality reads, using Cutadapt (version 5.0) [21] and Trimmomatic (version 0.39) [22], respectively, retaining high-quality reads (297 M reads) for further analysis. The quality of the trimmed reads was evaluated using FastQC (version 0.12.1). By using BWA (version 0.7.18) [23], the sequencing reads were mapped to the bovine reference genome (ARS-UCD 1.2) obtained from NCBI database [24]. The average mapping rate and the coverage for each chromosome were calculated using SAMtools (version 1.16.1) [25]. By using BCFtools (version 1.21) [26], a total of 8,434,516 variants were detected between the reference genome and the Japanese Black steer, which were used to construct a whole genome sequence of Japanese Black cattle of 2.7 Gb (Table S2A and S2B).

### Identification of known and candidate novel miRNAs

Both known and candidate novel miRNAs were identified from the small RNA-seq reads using miRDeep2 software package (Version 2.0.1.2) [27]. The small RNA-seq reads were mapped to the Japanese Black cattle whole genome. The mapped reads were sequentially annotated to bovine miRNA precursor and respective mature sequences obtained from the miRBase (version 22.1) [28], using the miRDeep2.pl module. Known miRNAs were identified from the perfectly annotated reads, while unannotated ones were modeled into precursor sequences, using RNAfold algorithm [27]. Candidate novel miRNAs were identified from unannotated reads whose precursor sequences could be folded into stable hairpin structures based on selection criteria of miRDeep2 scores ≥ 2 and Randfold *P* < 0.05. Genomic loci of the candidate novel miRNAs were confirmed against bovine reference genome (ARS-UCD2.0) using NCBI BLASTn search tools, according to the previous report [29]. Lastly, total read counts data of detected known and candidate novel miRNAs were calculated using quantifier.pl module, under the default settings.

### Analysis of plasma miRNA profiles and differential abundance

The miRNA abundance data and differential profiles were analyzed using edgeR package (Version 4.0.16) [30]. First, the total read counts were count-per-million (CPM) normalized, and miRNAs with CPM normalized counts greater than 5 in at least 3 of the 9 samples for each EX and FC fractions were selected for downstream analysis. Statistical differences in the number of the selected miRNAs between any two ages were tested using two-tailed paired t-test at *P* < 0.05. To assess the overall miRNA profiles among the 18 samples, Multidimensional scaling (MDS) plotting was performed with the plotMDS function in edgeR using log-transformed CPM values of the commonly identified miRNAs between EX and FC fractions, according to the previous recommendation [31].

Next, the trimmed mean of M values (TMM)-based normalization was performed to correct for possible miRNA compositional biases among the samples within the age groups [32]. Thereafter, paired test was performed under the default settings [30] to identify from all miRNAs differentially existed miRNAs (DE-miRNAs) among the age groups whose abundance varied with |log2 fold change|> 1 between two age groups at Benjamini and Hochberg's false discovery rate (FDR) < 0.05 [33].

### Reverse transcription-quantitative PCR (RT-qPCR) for DE-miRNAs

First strand cDNAs were synthesized from the miRNAs using the Mir-X miRNA First-Strand Synthesis Kit (Takara Bio Inc.), and then Quantitative PCR was performed using KOD SYBR™ qPCR Mix (TOYOBO) according to the manufacturers’ instructions. The reverse transcription and qPCR experiments were performed in MiniAmp Plus thermal cycler (Thermo Fisher Scientific) and Applied Biosystems StepOne Plus (Applied Biosystems), respectively. The forward primer sequences are shown in Table S3, while the common reverse primer was obtained from the First-Strand Synthesis Kit. The qPCR data were normalized to that of *bta-miR-93* whose abundance was relatively constant among the samples. Then, relative abundance of the candidate DE-miRNAs in plasma sample from 28 steers at 30 months of age was calculated using the 2-^ΔΔCT^ method [34].

### Correlation analysis of the DE-miRNA abundance and carcass traits

Carcass performance records were obtained for 28 steers at slaughter. The traits analyzed were cold carcass weight (CW), ribeye area (RA), rib thickness (RT), subcutaneous fat thickness (SFT), estimated yield percentage (YP), and marbling score (MS), according to the Japan Meat Grading Association [35]. Basic statistics of their carcass performance records are shown in Table 1. The Spearman’s Rank correlation coefficients among the DE-miRNA abundance and carcass traits of the 28 steers were calculated using *corr_coef* function in *metan* package (version 1.18.0) [36] and correlation plots were generated using *plot_cor* function in R language (version 4.4.1).

**Table 1:** Basic statistics on carcass performance records of 28 steers

Trait	Mean	SD	Minimum	Maximum
Cold carcass weight (kg)	533.0	64.3	404.2	690.7
Ribeye area (cm^2^)	62.3	8.5	52	80
Rib thickness (cm)	8.5	0.9	6.9	11.1
Subcutaneous fat thickness (cm)	2.8	0.7	1.6	4.0
Estimated yield percentage (%)	74.0	1.4	71.4	77.2
Marbling score	7.1	1.6	3	10

*SD* standard deviation

### Prediction of target genes of the DE-miRNAs

Putative target genes for the candidate DE-miRNAs were predicted using *miRWalk* database (version 3.0) [37] with criteria of sequence complimentarily of the miRNA “seed” with ≥ 1 and of the 3’ UTR region sequences at score ≥ 95%, according to the previous report [38]. Additional target genes were obtained through *TargetScan* database (version 8.0) [39].

### Gene ontology and Kyoto Encyclopedia of Genes and Genomes (KEGG) pathway enrichment analyses

Gene ontology (GO) analysis was performed for the list of putative target genes of miRNAs to predict the enriched biological processes (BP), cellular components (CC) and molecular functions (MF) using Database for Annotation, Visualization and Integrated Discovery (DAVID) functional annotation tool [40] under the default settings. In addition, Kyoto Encyclopedia of Genes and Genomes (KEGG) pathway enrichment analysis was performed for the list of putative target genes of miRNAs using clusterProfiler package [41] under the background genes from ‘org.Bt.eg.db’ database (version 3.18) [42].

### Statistical analysis

Statistical analyses were performed using R (version 4.4.1). Statistical differences in the number of the identified EX and FC miRNAs between any two ages were tested using two-tailed paired t-test. Differences were regarded as statistically significant if the *P* value was < 0.05.

## Results

### Reliability of the small RNA-seq reads and identification of plasma miRNAs

We obtained reliable sequencing reads with 99.99% sequencing confidence. The small RNA-seq reads quality and mapping results of all the 18 (9 EX and 9 FC) sequenced RNA samples are shown in (Table S4). The average optimum lengths of preprocessed reads were between 18–24 nucleotides, with the peak at 22 nucleotides (Fig. S1A and S1B), which characteristically indicates the lengths of mature miRNAs. On average, 43% and 71% of the reads from the EX and FC fractions, respectively, were mapped to the miRNA genomic regions (Table S4), corresponding to 1,030 known (Table S5) and 41 candidate novel miRNAs, respectively (Table S6). However, we found that *novel-miR-3* was the same as *bta-miR-2285au* (Fig. S2A). In addition, *novel-miR-30* was found to be a bovine ortholog of the known *miR-203a-3p* in other species such as humans (Fig. S2B); thus, it should be annotated as *bta-miR-203a-3p*. Accordingly, the remaining 39 miRNAs might be genuine novel miRNAs in cattle.

### Changes in plasma miRNA profiles during the fattening period

**Fig. 1:**
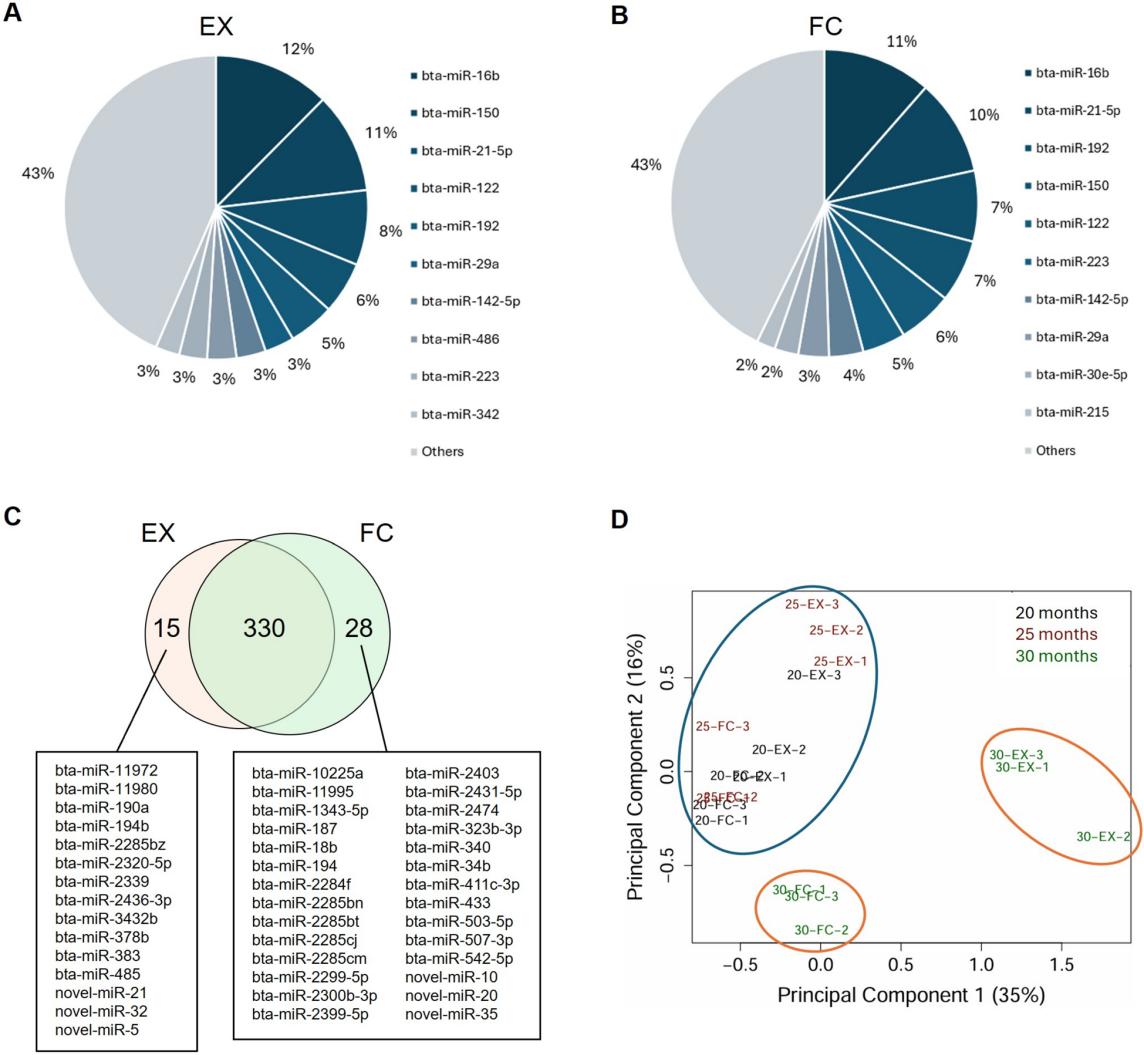
Plasma miRNA profiles during the fattening period. **A** and **B** Pie chart of the top 10 most abundant EX and FC miRNAs, respectively. **C** Venn diagram of EX and FC miRNAs whose CPM-normalized counts were > 5 in at least 3 samples of the respective plasma fractions. The lists show 15 and 28 miRNAs that exclusively existed in EX and FC fractions, respectively. **D** MDS plot based on the log of the CPM-normalized values of the 330 commonly existed miRNAs. The blue eclipse shows a single cluster at 20 and 25 months of age, while brown eclipses show two separate clusters for each of the EX and FC fractions at 30 months of age

Among a total of 1,071 miRNAs identified, 345 and 358 had CPM-normalized counts > 5 in at least 3 samples of the EX and FC fractions, respectively. The average CPM-normalized counts of the top 10 most abundant common miRNAs accounted for 57% of the total abundance for 345 and 358 miRNAs in EX and FC fractions, respectively (Fig. 1A and B). Among the top 10 abundant miRNAs, the existence level of *bta-miR-142-5p* and *−150* were greater at 20 and 25 months than 30 months of age, while that of *bta-miR-192* was greater at 30 months in both fractions (Tables S7 and S8). A total of 330 miRNAs (313 known and 17 candidate novel) were common in both fractions (Fig. 1C). The number of EX miRNAs showed a decreasing trend with fattening age in all the steers, with a significant reduction at 30 months of age (Fig. S3). The MDS plot showed three clusters; one including all samples at 20 and 25 months; one including all EX samples at 30 months; one including all FC samples at 30 months (Fig. 1D). This implies similar miRNA profiles among 20 and 25 months, which were collectively different with those of 30 months of age.

**Fig. 2:**
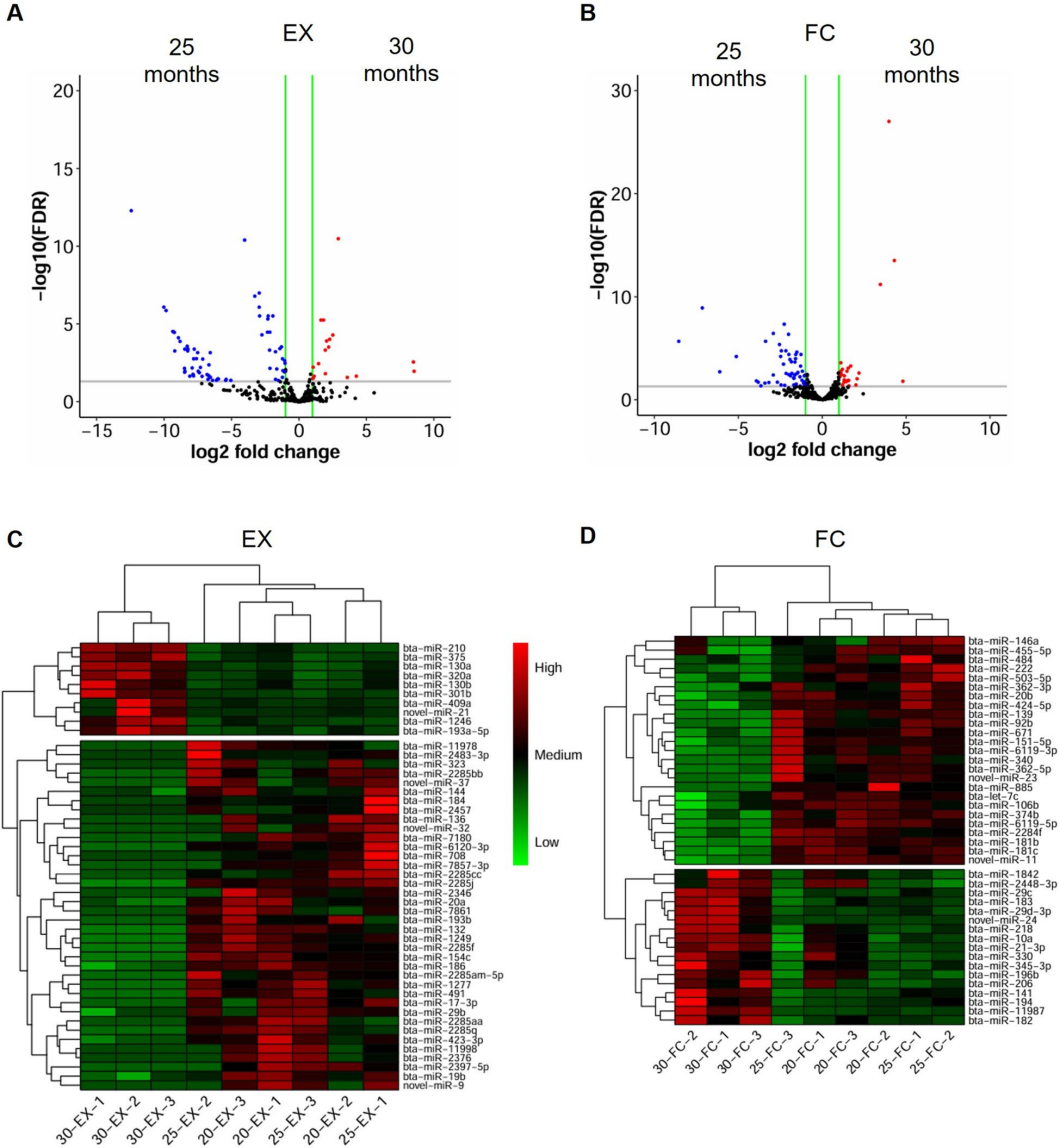
DE-miRNAs between 25 and 30 months of age. **A** and **B** The volcano plots for 86 and 80 DE-miRNAs in EX and FC fractions, respectively. Each dot represents a single miRNA, with red and blue dots indicating upregulated and downregulated DE-miRNAs at 30 months of age, respectively. Green horizontal lines indicate |log2 fold change|> 1, while grey horizontal ones, -log10 (FDR) ≥ 1.3 thresholds. **C** and **D** Hierarchical clustering of the EX and FC DE-miRNAs, respectively. Red and green colors represent higher and lower miRNA abundance at 30 months of age, compared to 20 and 25 months of age, respectively

We identified 86 EX and 80 FC DE-miRNAs between 25 and 30 months of age (Fig. 2A and B, Table S9). Hierarchical clustering of the EX and FC DE-miRNAs revealed single clusters at 30 months and collective ones at 20 and 25 months of age, respectively (Fig. 2C and D).

### qPCR quantification of the DE-miRNA abundance and correlation with carcass traits

**Fig. 3:**
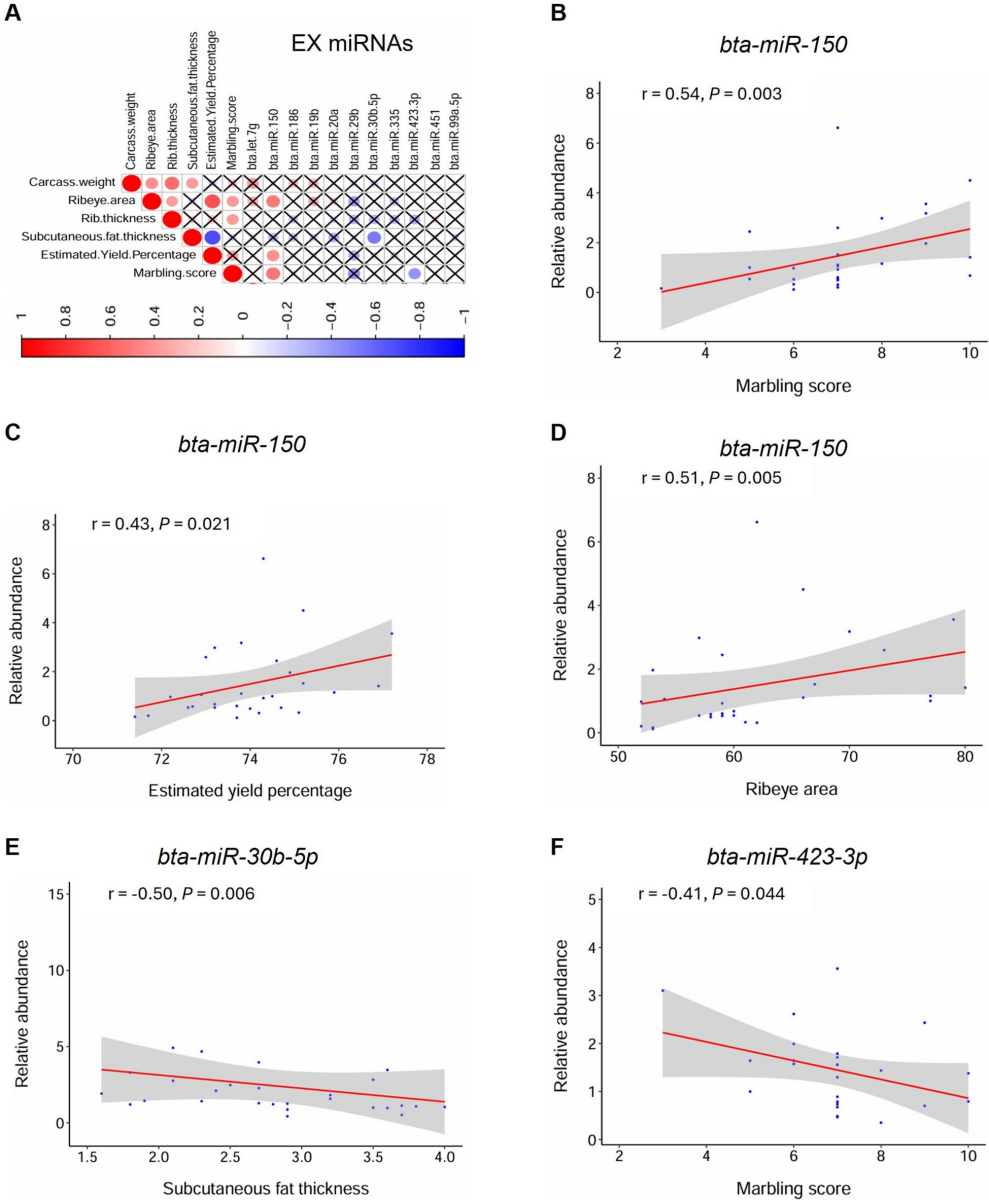
Correlation analysis of the abundance of EX miRNAs with the carcass traits. **A** Correlation heatmap with the circles showing the Spearman’s rank correlation coefficients. Red and blue colors represent positive and negative values, respectively, while sizes represent the magnitude of the correlations. ‘X’ crosses indicate nonsignificant correlations (*P* > 0.05). **B**, **C** and **D** Significant correlation plots of *bta-miR-150* with marbling score, estimated yield percentage, and ribeye area, respectively. **E** and **F** That of *bta-miR-30b-5p* and *−423-3p* and with subcutaneous fat thickness and marbling score, respectively. Y-axis shows the miRNA relative abundance, while the X-axis shows the measurement values of the carcass traits

**Fig. 4:**
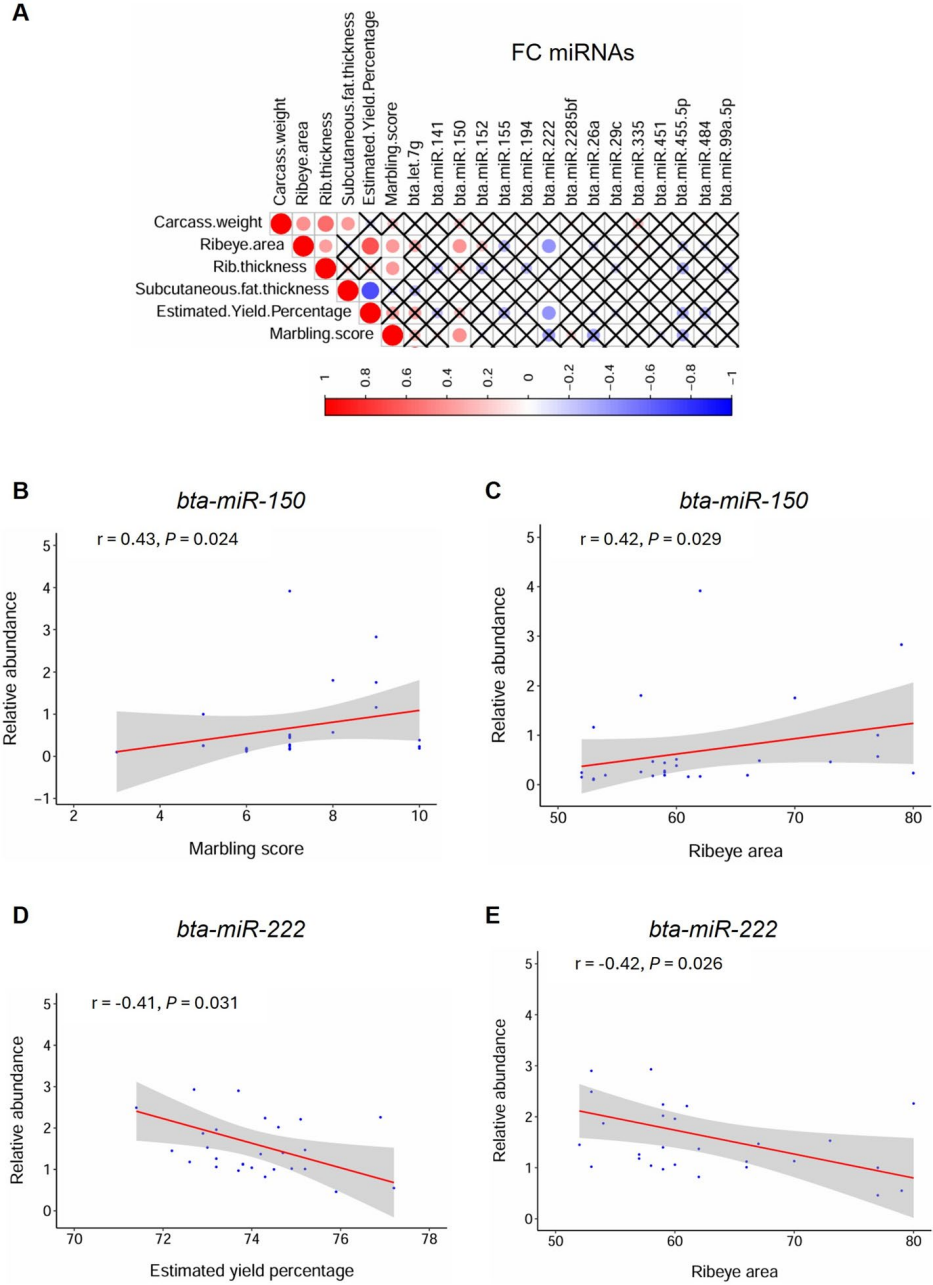
Correlation analysis of the abundance of FC miRNAs with the carcass traits. **A** Correlation heatmap with the circles showing the Spearman’s rank correlation coefficients. Red and blue colors represent positive and negative values, respectively, while sizes represent the magnitude of the correlations. ‘X’ crosses indicate nonsignificant correlations (*P* > 0.05). **B** and **C** Significant correlation plots of *bta-miR-150* with marbling score and ribeye area, respectively. **D** and **E** Significant correlation plots of *bta-miR-222* with estimated yield percentage and ribeye area, respectively. Y-axis shows the miRNA relative abundance, while the X-axis shows the measurement values of the carcass traits

Among the identified DE-miRNAs from the sRNA-seq analysis, 36 EX and 40 FC miRNAs had normalized abundance of > 50 counts in all samples of respective fraction at 30 months of age, and therefore they were subjected to qPCR analysis. As a result, 11 EX and 15 FC DE-miRNAs showed specific amplifications and were quantified in respective plasma fractions of the 28 Japanese Black steers at 30 months of age (Table S10). Among the EX miRNAs, the abundance of *bta-miR-150* showed significant correlations with MS (*r* = 0.54; *P* = 0.003), YP (*r* = 0.43; *P* = 0.021), and RA (*r* = 0.51; *P* = 0.005), while those of *bta-miR-30b-5p* and -*423-3p* with SF (*r* = –0.50; *P* = 0.006) and MS (*r* = –0.41; *P* = 0.044), respectively (Fig. 3A-F). Among the FC miRNAs, *bta-miR-150* significantly correlated with MS (*r* = 0.43; *P* = 0.024) and RA (*r* = 0.42; *P* = 0.029), while *bta-miR-222* with YP (*r* = –0.41; *P* = 0.031) and RA (*r* = –0.42; *P* = 0.026) (Fig. 4A-E).

### Functionally enriched GO terms and KEGG pathways for correlated miRNAs

**Fig. 5:**
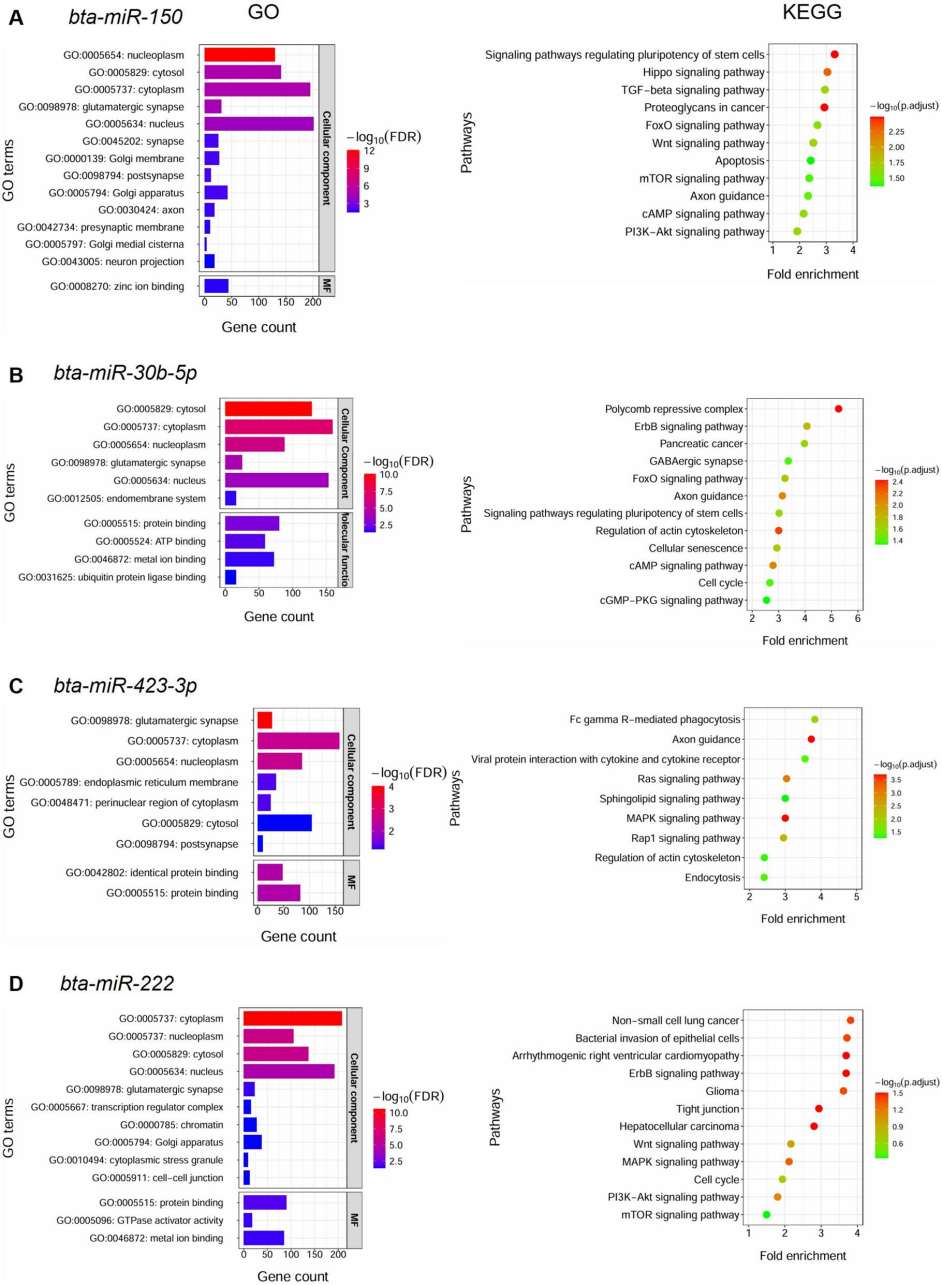
Functional enrichment analysis of the target genes of the correlated miRNAs. **A**, **B**, **C**, and **D** Significantly enriched GO terms (left) and KEGG pathways (right) for the target genes of *bta-miR-150, −30b-5p, −423-3p*, and *−222*, respectively. For GO terms, Y-axis shows the list of the enriched GO terms, while X-axis shows the number of genes involved in each GO term. For KEGG pathways, Y-axis shows the list of the enriched KEGG pathways, while X-axis shows fold enrichment which is defined as the percentage of genes belonging to the respective pathway, divided by the corresponding percentage in the background

We predicted 700, 502, 585, and 660 putative target genes for *bta-miR-150, −30b-5p*, *−423-3p,* and *−222*, respectively (Table S11), which were significantly enriched in 14, 10, 9, and 13 GO terms, and 11, 12, 9, and 12 KEGG pathways, respectively **(**Fig. 5A-D). The GO terms including nucleoplasm, cytosol, and cytoplasm were commonly enriched in the target genes for the correlated miRNAs. Signaling pathways such as PI3K-Akt, mTOR, and Wnt were commonly enriched in both *bta-miR-150* and *−222*. Signaling pathways regulating pluripotency of stem cells were common in both *bta-miR-150* and *−30b-5p*, while MAPK signaling pathway in both *bta-miR-222* and *−423-3p*.

## Discussion

We obtained high-quality small RNA-seq data corresponding to mature miRNAs from all the plasma samples, implying that the sequencing reads were reliable for extracellular miRNA profiling [43]. Nevertheless, we observed a low mapping rate for the EX fractions (43.6% on average) (Table S4), similar to previously reported mapping rate of 27–43% for EX miRNAs in humans [44]. We found a total of 1,071 miRNAs, including 41 potential candidate miRNAs. All the 41 potential candidate miRNAs had similar identities with the existing miRNAs, suggesting that they were from miRNA lineage. Among them, *novel-miR-3* has been revealed to be the same as *bta-miR-2285au*, having an SNP between Hereford and Japanese Black cattle (Fig. S2A). In addition, *novel-miR-30* was the same as *miR-203a-3p* in other mammalian species. Thus, *novel-miR-30* is now annotated as *bta-miR-203a-3p*. This is the first study to report on the existence of *bta-miR-203a-3p*. Lastly, *novel-miR-4, −6, −7, −13, −14, −17,* and *−39* had the same “seed” sequences as the known bovine miRNAs (Fig. S2C), implying that they belong to the existing miRNA families. MiRNA families have common structures and functions [45], thus, these candidate novel miRNAs may share physiological functions with the known miRNAs.

We identified 345 EX and 358 FC miRNAs which were present in measurable amounts in the plasma of Japanese Black steers during the fattening period. This is the first study to profile plasma EX and FC miRNAs separately in Japanese Black cattle. To date, it has been reported that microarray analysis of miRNAs from the plasma extracellular vesicles led to the detection of 202 miRNAs in fattened Japanese Black cattle [20]. Our data surpasses this in both quality and quantity. In Holstein–Friesian cross cows, small RNA-seq identified 386 [46] and 315 [47] miRNAs in plasma samples (> 10 reads per sample). In Holstein cows, small RNA-seq found 520 miRNAs in plasma, with 360 of those shared by all cows [17]. Our findings correspond well to the previous reports.

By correlation analyses using the 28 Japanese Black steers at 30 months of age, we found that the abundance of EX and FC *bta-miR-150* showed significant positive correlations with several carcass traits, such as MS, YP, and RA. Interestingly, *bta-miR-150* promotes differentiation of Qinchuan cattle-derived preadipocytes, possibly by modulating AKT1 phosphorylation within the PI3K/Akt/mTOR pathways [48]. In addition, high expression of *mmu-miR-150* has been linked to lipid metabolism in adipose tissue by modulating proinflammatory genes including *AdipoR2* and *Lep* in mice [49, 50]. These facts suggest that *bta-miR-150* may be associated with fat-related traits. The abundance of EX *bta-miR-30b-5p* negatively correlated with SF. *RGS2* (regulator of G protein signaling 2) is one of the target genes of *miR-30b-5p*, and *RGS2* knockout mice exhibit reduced weight and fat deposits [51], suggesting that *bta-miR-30b-5* may be associated with weight and fat-related traits. The abundance of EX *bta-miR-423-3p* showed a significant negative correlation with MS. Plasma level of *hsa-miR-423-3p* has been positively correlated with fat loss in subjects with type 2 diabetes [52], suggesting that *bta-miR-423-3p* may be associated with fat-related traits. The abundance of FC *bta-miR-222* negatively correlated with both YP and RA. *MiR-222* was strongly down-regulated upon differentiation of both primary and established myogenic cells [53], and overexpression of *miR-222* consequently silenced *Rbm24*, resulting in the inhibition of myoblast fusion in mouse satellite cells [54], indicating that *bta-miR-222* may be associated with muscle-related traits.

Despite moderate to high heritability estimates of carcass traits in Japanese Black cattle [55–58], carcass traits are highly polygenic and regulated by multiple polygenes. Thus, for the feasibility of using miRNAs as early indicators of carcass traits, multiple miRNAs should be identified to correlate to a single carcass trait. The overall predictive values of these miRNAs could then be determined by multiple regressions or predictive models based on machine learning algorithms. The candidate miRNAs identified in the present study would serve as fundamental data for future applications in deciding optimum slaughter age for individual Japanese Black fattened steers.

The present study had some notable limitations. Due to the small samples for the comprehensive analysis of plasma miRNA during the fattening periods (the same three individual samples for each time point), statistical power must be limited, and therefore chronological changes in the abundance of miRNAs have not been fully captured. In addition, correlation analyses using relatively small samples (28 individual samples) may miss several correlations of miRNAs with carcass traits. Therefore, larger samples collected from several different farms should be investigated in future study. Tissue-specific profiles including single cell RNA-seq analysis of the candidate miRNAs should also be performed to elucidate potential sources and mechanical insights of these miRNAs. Furthermore, in vitro functional analyses need to validate the potential roles of the miRNAs.

## Conclusions

In the present study, we investigated plasma EX and FC miRNA profiles during the fattening period in Japanese Black cattle. We found 1,071 miRNAs including 39 novel miRNA candidates. A total of 330 miRNAs were measurable and commonly detected in EX and FC fractions during the fattening period, the profiles of which were similar at 20 and 25 months, but collectively different at 30 months. We identified 86 and 80 DE-miRNAs between 25 and 30 months in EX and FC fractions, respectively. Among them, four miRNAs exhibited significant correlations with several carcass traits. Despite the limited number of samples analyzed, these results may support the usefulness of plasma miRNAs as potential early indicators of carcass traits in Japanese Black cattle.

## Supplementary Information

Supplementary Material 1: Fig. S1. Sequence length distribution of the small RNA-seq reads. Fig. S2. Comparison of known and candidate novel miRNAs. Fig. S3. Number of miRNAs present at different ages during the fattening period.

Supplementary Material 2: Table S1. Information for the three steers used for small RNA-seq.

Supplementary Material 3: Table S2. Results of whole-genome resequencing of Japanese Black cattle.

Supplementary Material 4: Table S3. List of 11 EX and 15 FC DE-miRNAs used for qRT-PCR analysis and their respective forward primer sequences.

Supplementary Material 5: Table S4. Small RNA-seq quality statistics and mapping results.

Supplementary Material 6: Table S5. List of miRNAs mapped to the Japanese Black cattle genome.

Supplementary Material 7: Table S6. List of 41 candidate novel miRNAs.

Supplementary Material 8: Table S7. Number of miRNAs whose CPM-normalized counts > 5 for at least 3 of all the 9 samples in each plasma fraction.

Supplementary Material 9: Table S8. Trend in the average abundance of the top 10 most abundant miRNAs at different ages.

Supplementary Material 10: Table S9. DE-miRNAs between 25 and 30 months of age.

Supplementary Material 11: Table S10. Basic statistics of qPCR quantification results.

Supplementary Material 12: Table S11. Target genes for bta-miR-150, -30b-5p, -423-3p, and -222.

## Data Availability

The data supporting the findings of this study are shown in this published article and its supplementary information files. All raw RNA sequencing data have been deposited in the DDBJ Sequence Read Archive (DRA; https://www.ddbj.nig.ac.jp/dra/index-e.html) under the accession number PRJDB20183. The processed data such as raw count data and CPM normalized count data have been deposited in the GEO repository (https://www.ncbi.nlm.nih.gov/geo/) under the accession number GSE319307. Since the whole genome sequencing data of Japanese Black cattle is property of the Japanese Black cattle producers in Japan and this information is commercially very sensitive, the data will be available upon reasonable request.

## Abbreviations

BP: Biological processes
BTA: Bos taurus
CC: Cellular components
CPM: Counts per million
CW: Cold carcass weight
DAVID: Database for Annotation, Visualization and Integrated Discovery
DE: Differentially existed
EX: Exosomal
FC: Non-exosomal free circulating
FDR: False discovery rate
GO: Gene ontology
KEGG: Kyoto Encyclopedia of Genes and Genomes
MF: Molecular functions
miRNA: MicroRNA
MDS: Multidimensional scaling
MS: Marbling score
QTL: Qualitative trait locus
RA: Ribeye area
RT: Rib thickness
RT-qPCR: Reverse transcription-quantitative PCR
SF: Subcutaneous fat thickness
RNA-seq: RNA sequencing
TMM: Trimmed mean of M values
YP: Estimated yield percentage
