# Supplementary material for: Comprehensive analysis of plasma exosomal and non-exosomal microRNA profiles identifies potential early indicators of carcass traits in Japanese Black cattle
Source: BMC Genomics. 2026 Apr 6;27:494. doi: 10.1186/s12864-026-12820-w (PMC13188637; doi:10.1186/s12864-026-12820-w)
Supplement: Supplementary file 1 — Supplementary Material 1: Fig. S1. Sequence length distribution of the small RNA-seq reads. Fig. S2. Comparison of known and candidate novel miRNAs. Fig. S3. Number of miRNAs present at different ages during the fattening period. [file 12864_2026_12820_MOESM1_ESM.pdf]

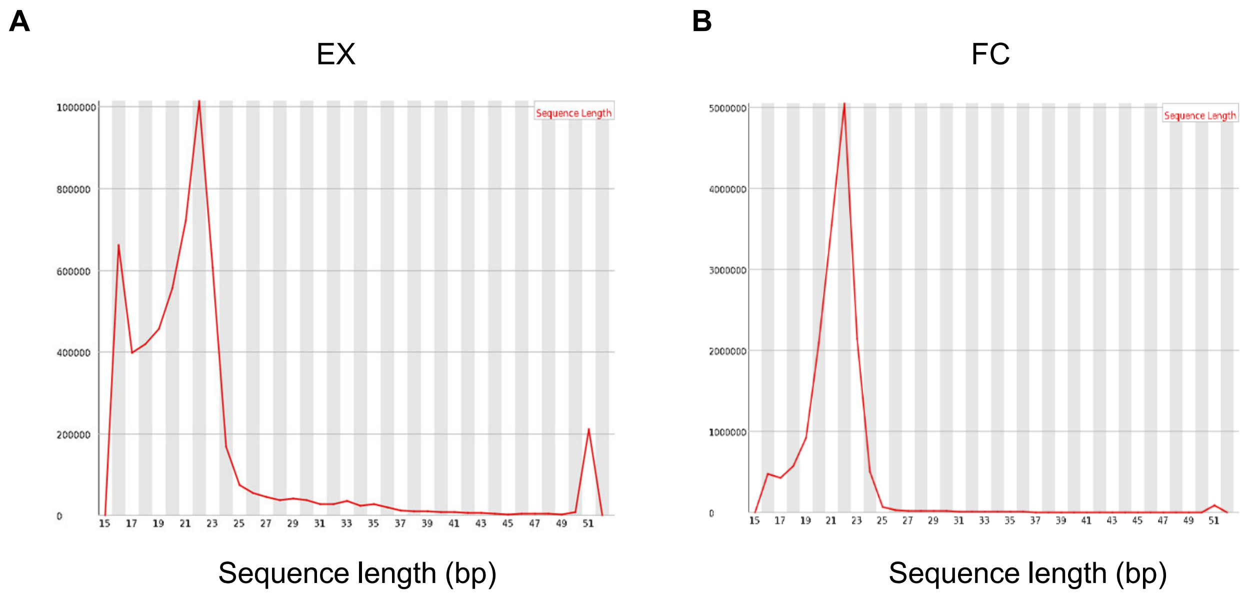

Fig. S1. Sequence length distribution of the small RNA-seq reads.
(A) and **(B)** Sequence length distribution of the sequencing reads for EX and FC miRNAs, respectively, showing the peak at 22 nucleotides. Representative data of the samples at 20 months of age were shown.

A Genomic region: BTA11, 60,611,028-60,611,082

novel-miR-3 **precursor**

AAAAAGUUCGUUUGGGUUUUCUgagccauguuacagaaaaacuugagugaacuuu

AAAAAGUUCGUUCGGGUUUUCUgagccauguuacagaaaaacuugagugaacuuu

bta-mir-2285au **precursor**

B Genomic region: BTA21, 68,645,107-68,645,168

novel-miR-30 precursor

aguggguucuuaacaguucaacaguucuguagcgcaauuGUGAAAUGUUUAGGACCACUAG

aguggguucuuaacaguucaacaguucuguagcgcaauuGUGAAAUGUUUAGGACCACUAG

hsa-miR-203a-3p **precursor**

C

(i)
novel-miR-4 - AAAAAGUUUGUUUGGGUUUU -
| | | | | | |
bta-miR-2284aa - AAAAAAGUUUGUUUGGGUUUU -

(ii)
novel-miR-6 - AAAACCCGAAUGAACUUUUUGG -
| | | | | | |
bta-miR-2285cx - AACACCCAAAUGAACUUUUUGA -

(iii)
novel-miR-7 - AAACCCACAUGAACUUUUUGG -
| | | | | | |
bta-miR-2285cx - AACACCCAAAUGAACUUUUUGA -

(iv)
novel-miR-13 - AUAAAUUCGUUUGGAUUUUUCC -
| | | | | | |
bta-miR-2284e - AAGUUCGUUCGGAUUUUUCC -

(v)
novel-miR-14 - AUAAUACAACCUGAUAAGU -
| | | | | | |
bta-miR-374c - UUACAAUACAACCUGAUAAGU -

(vi)
novel-miR-17 - GAAAUCCGAACGAACUUUUUGG -
| | | | | | |
bta-miR-2285i - AAAACCGGAACGAACUUUUUGG -

(vii)
novel-miR-39 - GAAAAAGUUUGUUUGGGUUUUU -
| | | | | | |
bta-miR-2284z - AAAAAAGUUUGUUUGGGUUUUU -

Fig. S2. Comparison of known and candidate novel miRNAs.
(A) Genome context of *novel-miR-3* and *bta-miR-2285au* precursor sequences. The red rectangle shows the location of a SNP on mature (capital letters) sequences. **(B)** Genome context of *novel-miR-30* and *hsa-miR-203a-3p* precursor sequences. **(C)** Identical "seed" sequences (red letters) of *novel-miR-4, 6, 7, 13, 14, 17,* and *39* with those of the respective known miRNAs.

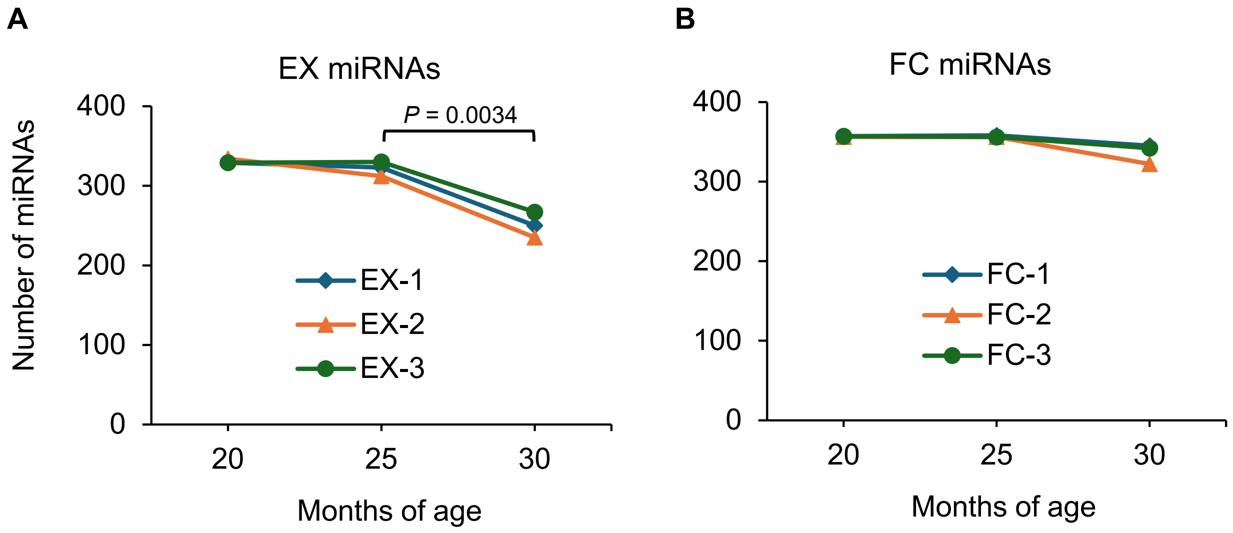

Fig. S3. Number of miRNAs present at different ages during the fattening period.
(A) and **(B)** Line graph showing the trend in the number of EX and FC miRNAs, respectively, present in each steer at different ages during the fattening period. The number of EX miRNAs was significantly reduced at 30 months of age.
